# Another Chest Pain Ruled Out: A Typical Story With an Atypical Finding on a Chest X-ray (CXR) Leading to an Uncommon Diagnosis

**DOI:** 10.7759/cureus.80668

**Published:** 2025-03-16

**Authors:** Daniel Spector, Kevin Parsons, Megan Stobart-Gallagher

**Affiliations:** 1 Emergency Medicine, Jefferson Einstein Montgomery Hospital, East Norriton, USA; 2 Radiology, Jefferson Einstein Philadelphia Hospital, Philadelphia, USA

**Keywords:** abnormal xray, atypical chest pain, emergency medicine, pleuritic, pulmonary embolism mimic

## Abstract

Epicardial fat necrosis is an uncommon cause of chest pain that mimics pericarditis, pulmonary embolism, and/or coronary syndrome. This is a case of a 55-year-old female patient presenting with left-sided pleuritic chest pain. She had a normal exam and vital signs. Diagnostics showed a non-ischemic electrocardiogram with a negative troponin, an elevated D-dimer, and a chest radiograph showed an opacity along the left cardiac border. Computed tomography-pulmonary angiography (CT-PA) was performed and showed a 4.2 x 1.8 x 3.0 cm lesion, surrounded by a hyperdense ring of high attenuation with fat stranding, which diagnosed epicardial fat necrosis.

## Introduction

Necrosis of the adipose tissue surrounding the heart is a rare cause of acute chest pain, but it should be kept in the differential diagnosis of emergency physicians taking care of acute chest pain patients. Epicardial fat necrosis is self-limiting and overall a benign cause of chest pain [1]. It was first described in 1957 by Jackson et al. in a series of three cases. In these cases, the area of fat necrosis was concerning for an underlying malignancy and was removed surgically [2]. Epicardial fat necrosis remains relatively rare, with an adolescent case report describing <50 cases documented as of 2021 [3]. Until recently, there was no recommended algorithmic approach to investigation and management.

Chest pain is a very common presenting concern from patients who present to the emergency department (ED) and can often indicate underlying life-threatening conditions such as myocardial infarction, myocarditis, pulmonary embolism, or aortic dissection, to name a few. A patient with epicardial fat necrosis may also present with chest pain, but fortunately, it is a benign, self-limiting condition. Most likely, this diagnosis will be found on the basis of ruling out life-threatening causes of chest pain. Most cases occur in young, healthy adolescent patients [3]. We present a case of epicardial fat necrosis occurring in a 55-year-old female with comorbidities that put her at risk for a potentially serious diagnosis.

## Case presentation

A 55-year-old female presented to the emergency department (ED) with an abrupt onset of 36 hours of left-sided pleuritic chest pain. The pain worsens when taking deep breaths and leaning forward at the waist. It improves with sitting straight up and is not reproducible to palpation. On review of systems, she reports a transient episode of left calf pain a week prior to presentation and has had intermittent bilateral lower extremity edema that resolves with elevation. She has had no recent periods of immobilization, has no history of trauma, and has no medical history to prompt concern for an underlying hypercoagulable state. She has a past medical history of hypertension, obstructive sleep apnea for which she wears nightly CPAP, hyperlipidemia, allergic rhinitis, and hypothyroidism, which are all under routine care of a primary care physician. She does have a family history of ischemic cardiac disease. Her medications are as follows: albuterol inhaler, alprazolam, cetirizine, levothyroxine, montelukast, and triamcinolone acetonide nasal spray.

On physical examination, her oral temperature was 37.6°C, heart rate 85 beats per minute, blood pressure 139/61 mm Hg, respiratory rate 18 breaths per minute, and oxygen saturation of 98% on room air. Her cardiovascular examination was without murmurs, rubs, or gaps. She had no rashes overlying the impacted area and no reproducible tenderness to palpation along the chest wall. She did point to the area underneath her left breast as the impacted area of worst pain, especially while taking a deep breath. Her lower extremities were warm and well-perfused with equal palpable dorsalis pedis pulses without asymmetry or pitting edema. She had no Homan’s sign. Her lungs were clear to auscultation bilaterally and she had no increased work of breathing. 

**Figure 1:**
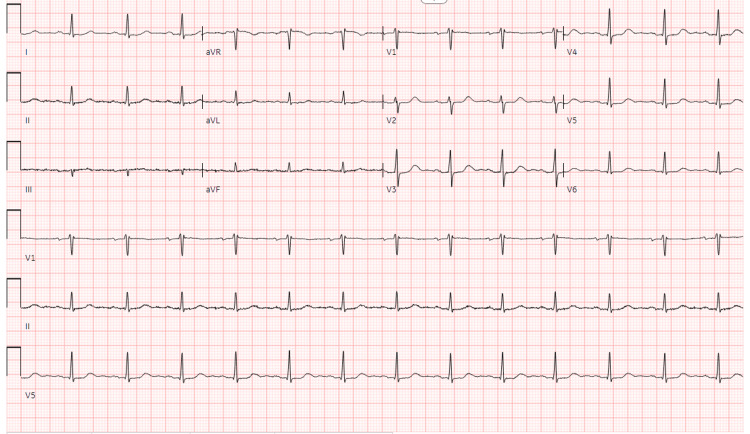
Electrocardiogram showing normal sinus rhythm at 80 beats per minute with normal intervals and no signs of acute occlusive disease.

The initial ED differential diagnosis included acute coronary syndrome (ACS), pulmonary embolism, pneumonia, pericarditis, or musculoskeletal chest pain. The patient was given 324 mg of aspirin to treat for potential atypical acute coronary syndrome as well as for analgesia. An EKG was performed that showed sinus rhythm at 80 bpm with normal intervals and no signs of occlusive myocardial infarction (Figure 1). 

**Figure 2:**
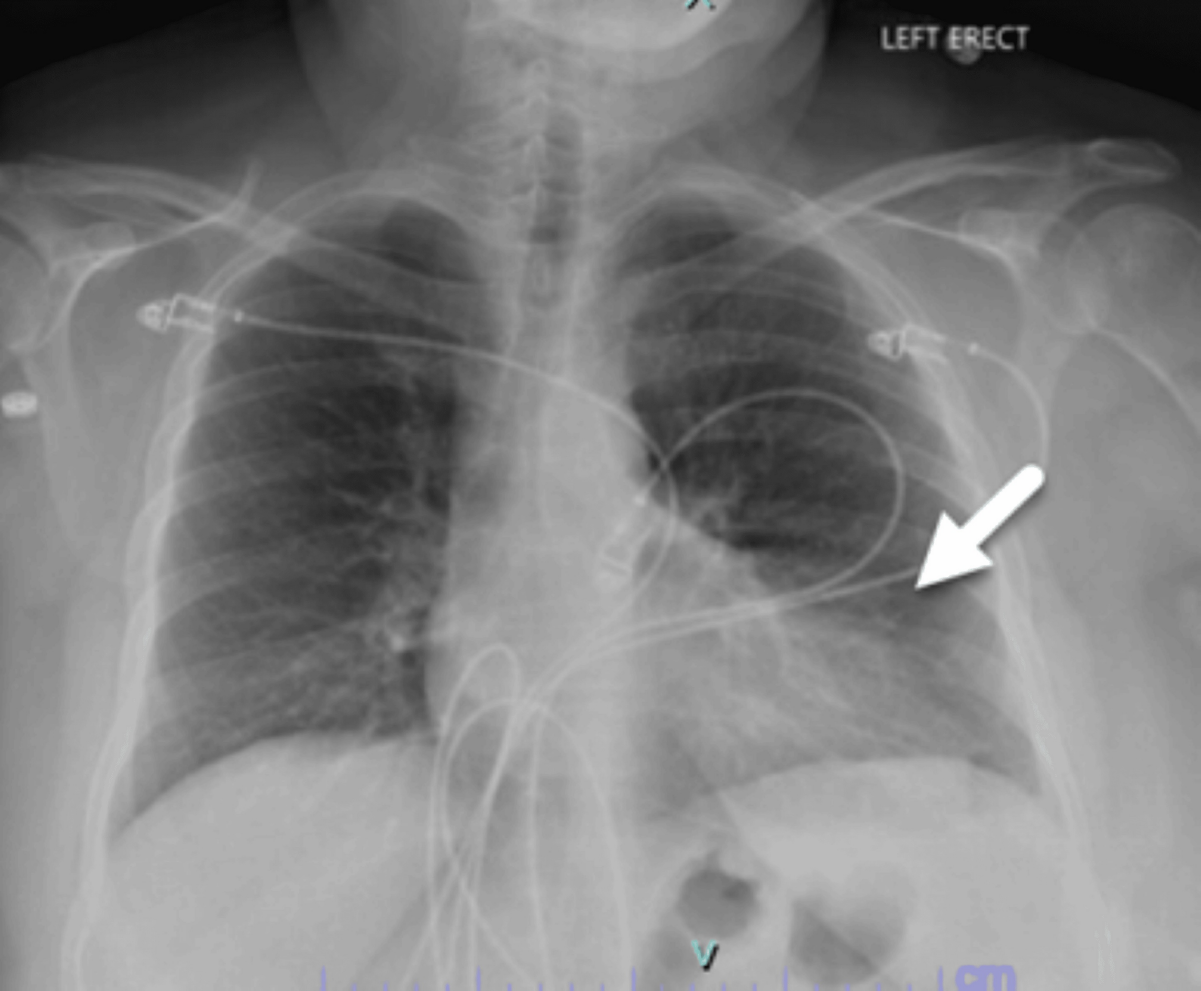
Chest radiograph showing opacity along the left cardiac border.

Blood work performed showed normal hemoglobin, hematocrit, and platelets. She had a very mildly elevated creatinine at 1.27 mg/dL but normal electrolytes, including sodium, potassium, chloride, and calcium levels. Her troponin was <0.01 ng/mL. Based upon her Wells Score for probability of pulmonary embolism (low risk category), a D-dimer was performed and was elevated at 681 ng/mL (<400 ng/mL normal range). A chest radiograph was performed that showed no acute pneumothorax or consolidation, but noted by radiology was an indistinctness of the left cardiac border of unclear etiology (Figure 2). 

**Figure 3:**
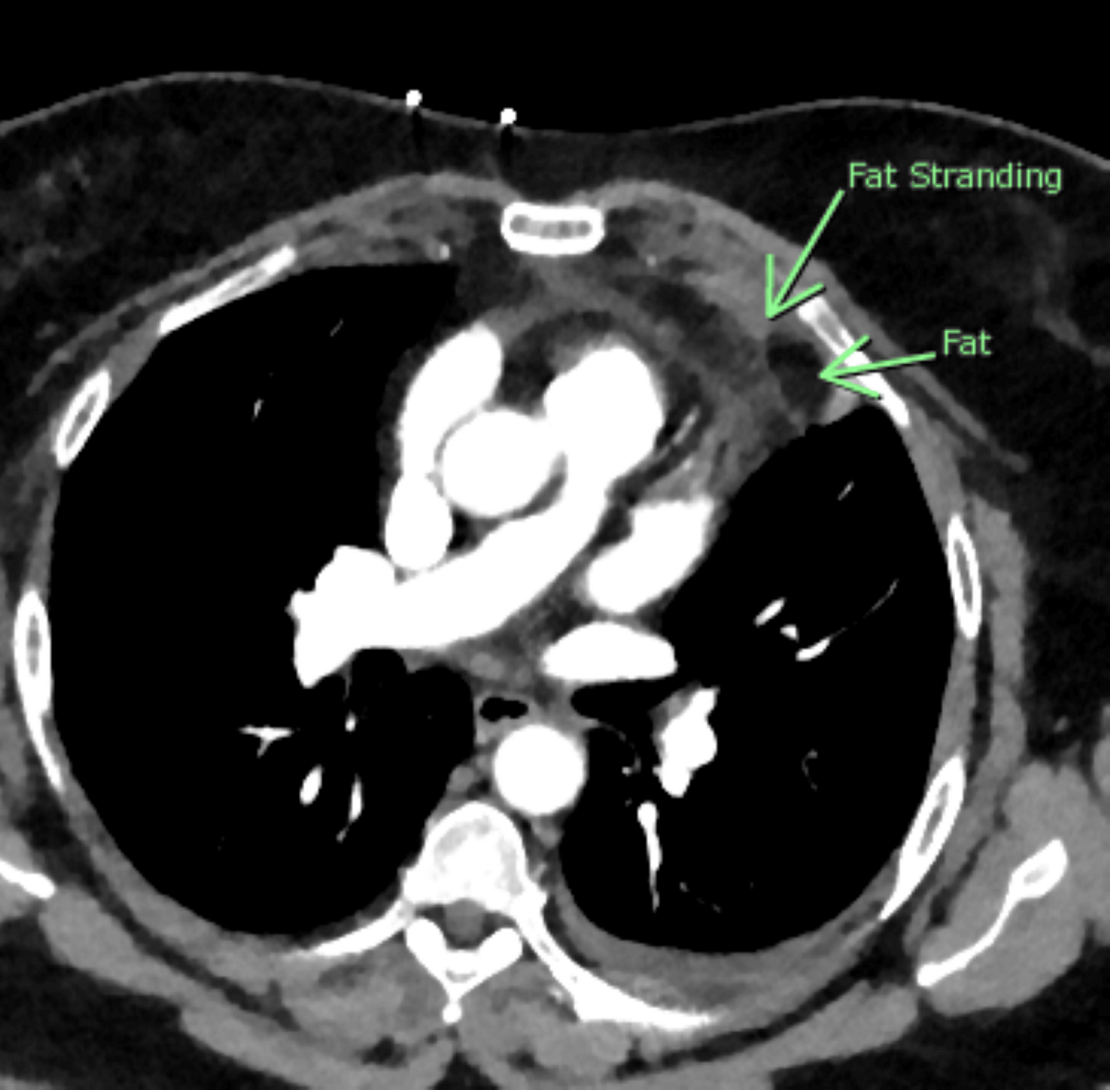
Axial view of CT showing a 4.2 cm x 1.8 cm x 3 cm lesion in the left epicardial space, surrounded by an area of adjacent fat stranding suggestive of central necrosis.

**Figure 4:**
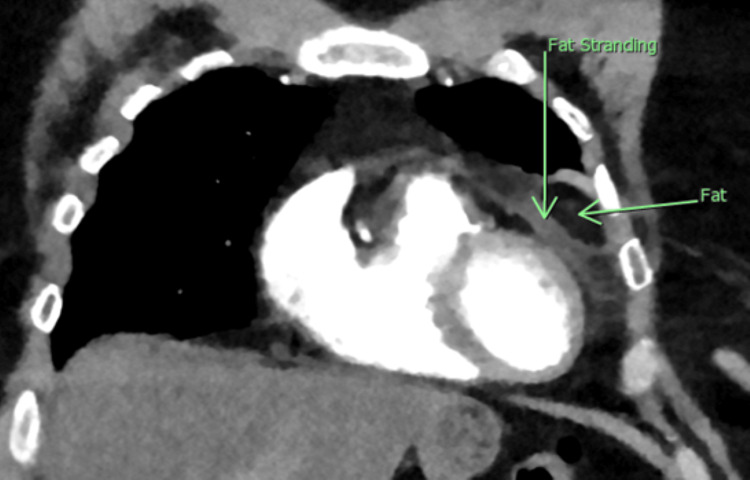
Coronal image from computed tomography showing a 4.2 cm x 1.8 cm x 3 cm lesion within the epicardial space using green arrows to differentiate the area of necrosis and surrounding fat stranding.

Based on the elevated D-dimer and abnormal chest radiograph, the diagnosis of pulmonary embolism was pursued with computed tomography (CT). The CT scan showed a 4.2 cm x 1.8 cm x 3 cm fat attenuating lesion in the left epipericardial/pericardial fat pad, surrounded by a hyperdense ring of high attenuation with adjacent fat stranding in the surrounding fat concerning for acute epipericardial/pericardial fat necrosis. There was also a trace amount of associated adjacent pericardial thickening/effusion that was thought to be reactive in nature (Figures 3, 4).

Our patient was informed of the uncommon diagnosis and treated with intravenous nonsteroidal medications. Her pain improved in real time, and she was discharged with anti-inflammatory medications and outpatient follow-up. She followed up with her primary care physician within three months and had complete resolution of symptoms.

## Discussion

Chest pain remains a common presentation for ED visits across the United States and encompasses all age ranges. As risk and concern for life-threatening conditions are the predominant indication for a diagnostic workup in the ED, epicardial fat necrosis is almost always found as an incidental cause. It is thought to be due to a spontaneous torsion of the vascular pedicle or a capillary rupture within the thoracic cavity [4]. This may occur due to a sudden Valsalva maneuver or overtime with frequent bearing down, coughing, or sneezing. As epicardial fat necrosis remains mostly described as an incidental finding, many emergency physicians may not be aware of this diagnosis, which could lead to multiple ED visits [5].

Epicardial fat necrosis typically presents with sharp pain localized over the left shoulder or within the chest cavity, often pleuritic in nature. Physical examination is often completely normal, but patients could have an elevated heart rate, respiratory rate, or even reproducible pain with movement [1]. Electrocardiograms for patients are almost always normal, except for the occasional nonspecific repolarization finding, and chest radiographs can show some abnormalities but in less than 5% of diagnoses. There is currently no literature to support the use of point of care ultrasound as a diagnostic tool for this diagnosis. Formal transthoracic echocardiography can detect hypoechogenic areas overlying the pericardium and left ventricle [6], but that is not within the recommended scope of practice for emergency physicians at this time [7].

Chest radiographs can be nonspecific with atelectasis, a small left-sided pleural effusion, pericardiac opacity, or pericardial thickening [8]. These chest radiograph findings may prompt additional diagnostic testing, which often leads to a CT scan, discovering the diagnosis. A noncontract CT scan of the chest is considered the gold standard for this diagnosis and shows pericardial thickening with an encapsulated fatty lesion [9]. A retrospective review from 2021 of chest CTs (n=7463) found the presence of epicardial fat necrosis in 2.15% of scans. Thus, both radiology and emergency medicine physicians should be aware of this condition and look for it when reviewing imaging in specific clinical scenarios. It is important to note that the differential for a pericardial fatty lesion includes primary fatty tumors such as lipoma, liposarcoma, teratoma, and thymolipoma [10], so repeat imaging will be incredibly important for patients to assess for resolution.

Accorsi et al. proposed a diagnostic algorithm for the approach and management of presumed epicardial fat pad necrosis, which recommends a reasonable approach to work-up for acute pleuritic chest pain depending upon the patient’s risk factors. If pleuritic chest pain is present, a thorough history and physical examination should be performed. Electrocardiography and chest radiography should be considered at a minimum if epicardial fat necrosis is suspected. Additional tests should be included in the diagnostic approach if other conditions are suggested by the patient's history and risk factors. If no definitive diagnosis is established, a radiology-performed transthoracic echocardiogram or chest CT-PA should be considered to rule out epicardial fat necrosis [1].

The treatment for epicardial fat necrosis is anti-inflammatory medications and a one to two-month follow-up with repeat imaging to ensure resolution because it is important to remember that if there is no resolution, this fatty lesion could be representative of malignancy [9]. This information is very important to include in any discharge instructions for the patient.

## Conclusions

While chest pain is a common presenting concern to the ED, the diagnosis of epicardial fat necrosis is uncommon. This should be suspected in patients with unexplained pleuritic or precordial chest pain that have a pericardial opacity on chest radiograph. Diagnosis can then be confirmed through a transthoracic echocardiogram or a CT-PA. These patients can subsequently be treated with anti-inflammatory medications as outpatients with instructions to obtain follow-up imaging to ensure resolution.
